# Generation of 99.8 fs, 25 kW Peak-Power, Dispersion-Managed Pulses Directly from an Yb-Doped Figure-of-9 Fiber Laser

**DOI:** 10.3390/ma15197038

**Published:** 2022-10-10

**Authors:** Shuai Yuan, Lu Si, Jianing Chen, Junyu Chen, Han Yu

**Affiliations:** 1 Shanghai Key Lab of Modern Optical System, and Engineering Research Center of Optical Instrument and System, Ministry of Education, School of Optical Electrical and Computer Engineering, University of Shanghai for Science and Technology, Shanghai 200093, China; 2 Chongqing Key Laboratory of Precision Optics, Chongqing Institute of East China Normal University, Chongqing 401120, China; 3 State Key Laboratory of Precision Spectroscopy, East China Normal University, Shanghai 200062, China; 4 Jinan Institute of Quantum Technology, Jinan 250101, China

**Keywords:** fiber lasers, mode-locked lasers, ultrashort pulse lasers, ytterbium fiber

## Abstract

We reported on the generation of 99.8 fs, 25 kW peak-power, dispersion-managed pulses directly from a passively mode-locked Yb-fiber laser oscillator with a figure-of-9 configuration. The introduction of strongly injected pump power and optical components with a high damage threshold enables high-power operation, while the polarization-maintaining (PM) fiber supports environmentally stable self-started mode-locking. Mode-locking in the soliton-like and negative-dispersion regime is characterized by the dispersion management via tuning the separation distances between a pair of gratings inside the cavity. The oscillator generates stable pulses with up to 40.10 mW average power at a 16.03 MHz repetition rate, corresponding to a pulse energy of 2.5 nJ. To the best of our knowledge, it is the highest peak-power directly obtained by a laser oscillator with a figure-of-9 configuration.

## 1. Introduction

Ultrashort pulse oscillators in the Yb-gain spectral region, around 1 μm, routinely drive high-power Yb-amplifiers, powering the growing number of ultrashort pulse applications that require stability against environmental perturbations, high pulse energies, and short pulse durations [1,2]. In this sense, fiber lasers mode-locked by means of a nonlinear amplifying loop mirror (NALM) have caught considerable attention in recent years owing to a relatively high damage threshold and excellent flexibility [3,4]. As a fiber-nonlinearity-based artificial saturable absorber, the mode-locking method of NALM is based on the intensity-dependent transmission of the Sagnac fiber loop.

Compared with nonlinear polarization evolution (NPE), NALM has the advantage of mode-locking stability and lower intrinsic noise since it does not rely on polarization rotation and can be implemented in PM format [5,6,7,8]. In spite of the simplicity and stability, a fatal shortcoming for lasers based on NALM is their poor self-started ability [9]. To overcome the drawback, a fiber laser with a figure-of-9 configuration was proposed, including a single loop and a linear arm [10,11]. The scheme has been named “figure-of-9” laser due to the straight arm. On the other hand, the use of a phase shifter releases the requirement for nonlinear phase shifts and reduces the mode-locking threshold [12]. With the introduction of the figure-of-9 configuration and phase shifter, the self-started ability of NALM was greatly optimized [9]. In 2020, M. Edelmann et al. numerically and experimentally generated transform-limited, 64 fs pulses with 10 kW peak-power directly from the output of a modified Yb-doped figure-of-9 fiber laser by overdriving the transmittance of the NALM [13]. More recently, H. Chu et al. achieved a dispersion-managed figure-of-9 configuration by using the polarization beam combiner and a grating pair. The direct output pulse had a peak-power of 4.85 kW and a pulse duration of 122 fs [14].

Although significant steps have been made, lasers based on a figure-of-9 configuration have not generated more than 0.61 nJ and shorter than 100 fs pulses, with the average power limited to 21 milliwatts [14]. As shown in Table 1, we compare this work with other published studies. The results show that the power scaling potential of the figure-of-9 configuration remains to be explored. In addition, compared with de-chirping outside the cavity, intra-cavity dispersion management can simplify the laser system and reduce the cost of the laser significantly [15,16,17,18,19,20,21]. Among the dispersion management elements, the grating pair has the advantage of shortening the cavity length and flexible dispersion adjustment [22,23,24,25].

In this paper, we demonstrate an all PM dispersion-managed mode-locked laser oscillator with a figure-of-9 configuration, which generates 25 kW peak-power pulses at 1.03 μm. The high peak-power delivered directly from the oscillator was attributed to the combination of higher injection power and the designed collimator. With stable operation and self-started mode-locking, the central wavelength of the laser output could be tuned from 1029 to 1042 nm by controlling the inter-cavity dispersion. In our experiment, a −π/2 phase shifter was inserted in the nonlinear loop to reduce the mode-locked threshold, and a compactible grating pair was employed in the linear arm to manage the inter-cavity dispersion. With an optimized separation of the grating pair, the pulse duration was achieved as short as 99.8 fs. The highest peak-power of the direct output was 25 kW, the pulse energy 2.5 nJ, with 12.06 nm for the corresponding spectral width.

## 2. Experimental Setup

**Figure 1:**
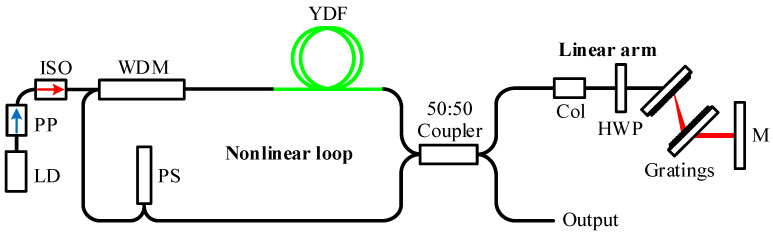
Schematic configuration of the laser structure. LD: 900 mW laser diode; PP: pump protector; ISO: isolator; WDM: wavelength division multiplexer; YDF: Yb-doped fiber; Col: collimator; HWP: half wave plate; M: reflect mirror; PS: phase shifter.

Figure 1 shows the schematic of the experimental setup of dispersion-managed with a figure-of-9 configuration. The laser cavity consisted of an 8.1 m long nonlinear loop and a 1.75 m long linear arm. The nonlinear loop included a wavelength division multiplexer (WDM), a 1.3 m Yb-doped fiber (PM-YSF-HI-HP, NUFERN, East Granby, CT, USA), and a phase shifter (AFRLASER, Zhuhai, China) in a sequence. The Yb-doped fiber as the gain medium was asymmetrically located in the nonlinear loop, with 0.5 m fiber pigtails between the WDM. In this sense, the differential nonlinear phase shift was yielded for pulses oscillating along opposite directions. The Yb-doped fiber was pumped by a 976 nm laser diode (LD) (3SP Technologies, Nozay, France) with a maximum pump power of 900 mW. The injected power from the pump diode was higher compared with previous experiments based on the figure-of-9 configuration [14]. A pump protector (AFRLASER, Zhuhai, China) and an isolator (AFRLASER, Zhuhai, China) with an operating power of 1 W were placed sequentially behind the LD, which prevented the LD from being damaged due to back reflections. A reflection-type phase shifter working at 1030 nm was employed to provide a non-reciprocal phase shift of −π/2. The phase shifter was a commercial one, which consisted of a polarization beam splitter (PBS), a Faraday rotator, a λ/8 waveplate, and a mirror. The linear arm included a collimator, a half-wave plate, a transmission grating pair, and a high reflection mirror. In our attempt, the fiber tip in the collimator was easily damaged due to the high pump power. We solved this problem by enlarging the fiber tip inside the collimator using a silica fiber (AFRLASER, Zhuhai, China) with a 125 μm diameter. The polarization of the beam exiting the collimator was rotated by the half-wave plate in order to satisfy the polarization for the maximum transmission of the grating pair. The high reflection mirror was employed after the grating pair to reflect the beam back into the fiber loop. A pair of 1250 line/mm transmission gratings (EACHWAVE, Shanghai, China) were arranged for intra-cavity dispersion compensation. The grating pair was mounted on translation stages for dispersion management. In our experiment, dispersion management was performed by adjusting the separation between the grating pair. The nonlinear loop and linear arm were connected by a 2 × 2 coupler (AFRLASER, Zhuhai, China) with a 50:50 splitting ratio, which acted as the entry/exit point of the loop. The oscillating pulse was exported from the cavity by the other port of the coupler. The interference of the counter-propagating pulses at the coupler resulted in an intensity-dependent transmission, forming an artificial saturable absorber to lock the pulse. All the fibers inside the cavity were single-mode PM fiber.

## 3. Results and Discussion

**Figure 2:**
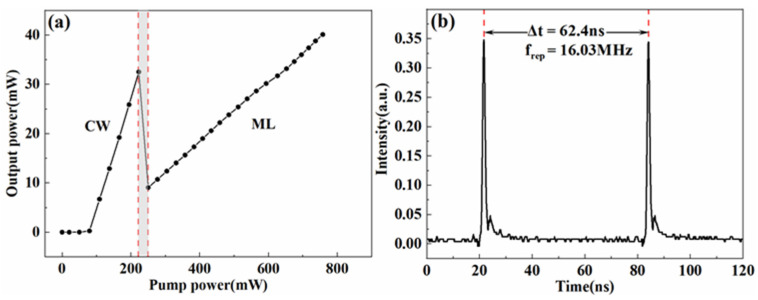
(**a**) Experimentally measured output powers with respect to different pump powers. CW: continuous wave; ML: mode-locking. (**b**) The pulse train generated from the fiber oscillator.

Figure 2a shows the output powers under different pump powers. The output powers were recorded at the output port of Figure 1 by a power meter (THORLABS, PM100D, Newton, NJ, USA). When we gradually increased the pump power from 220 mW to 250 mW, the output became a Q-switched state from a continuous-wave (CW) state (see the gray region in Figure 2a). As the pump power exceeded 250 mW, a reliable self-started operation was easily achieved. With higher pump power, the tips of the fiber at the collimator were easily damaged. Thus, in our experiment, we did not tune the power of the pump beyond 750 mW. The achieved highest output power was 40.10 mW under the 750 mW for the pump power, which corresponded to the pulse energy of 2.50 nJ. The state of the mode-locking would vanish when the pump power was lower than 178 mW. The output pulse train was recorded by a 2.5 GHz photodetector (LIGHTSENSIN, LSIPD-A75, Beijing, China) and an oscilloscope (RIGOL, MSO2302A, Beijing, China), as shown in Figure 2b. The laser pulses had a repetition rate of 16.03 MHz, which essentially agreed with the calculation from the cavity length. The pulse train exhibited high stability, and no satellite pulses were found within the two adjacent pulses.

**Figure 3:**
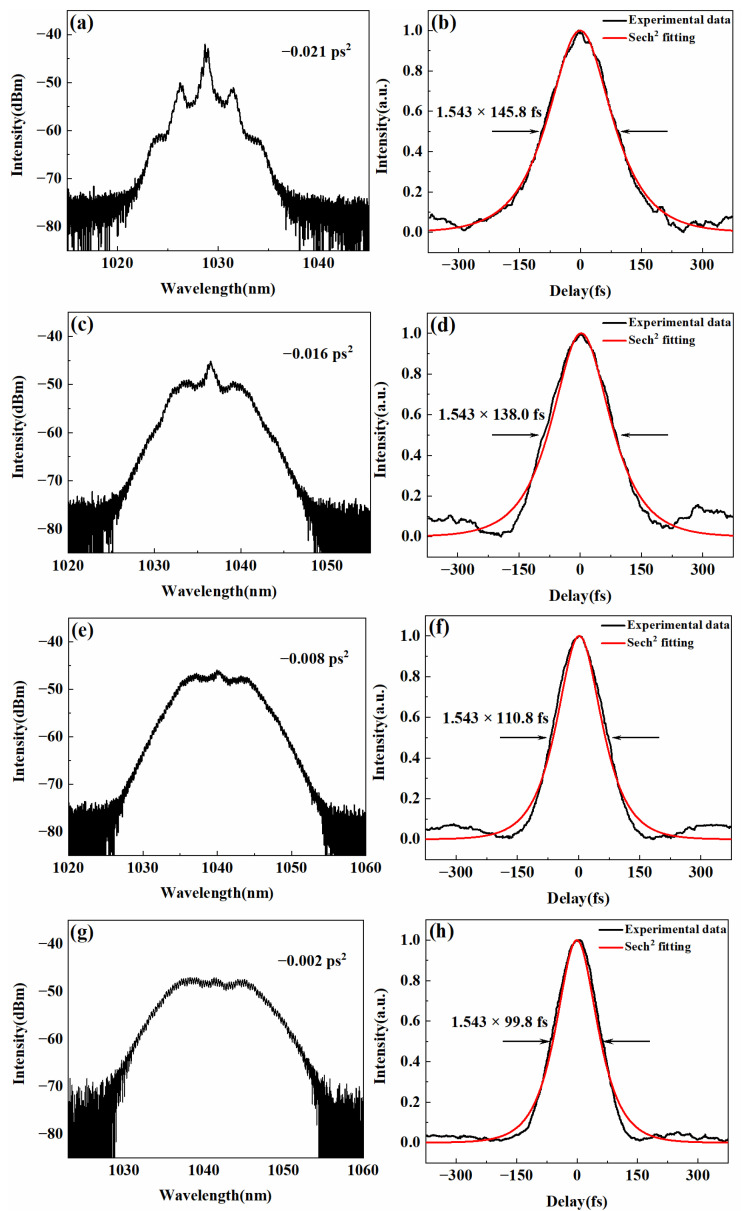
The evolution of optical spectra and pulse duration with the net inter-cavity dispersion tuning from −0.021 ps^2^ to −0.002 ps^2^. The measured spectra are presented in logarithmic scales in (**a**,**c**,**e**,**g**). The corresponding autocorrelation traces of experimental (black) and sech^2^ fitting (red) pulses are shown in (**b**,**d**,**f**,**h**).

In order to manage the inter-cavity dispersion, we investigated the laser operation with different grating separations. The positive group delay dispersion (GDD) in the cavity was contributed by 9.7-m long fibers. The GDD was calculated to be 0.25 ps^2^ by considering 26 fs^2^/mm for the group velocity dispersion (GVD) of the fibers. While the negative dispersion was attributed to the grating pair, whose GVD was −2.68 × 10^4^ fs^2^/mm. The output spectra and pulse duration were analyzed in Figure 3, with a pump power of 750 mW. The optical spectra were monitored by an optical spectral analyzer (THORLABS, OSA202C, Newton, MA, USA) with a minimum resolution of 0.02 nm. The autocorrelation trace was measured by an intensity autocorrelator (PulseCheck50 NIR, APE, Berlin, Germany). The mode-locking was maintained as the grating pair separations were tuned from 10.2 to 9.5 mm, corresponding to net cavity dispersions of −0.021 to −0.002 ps^2^.

Figure 3a,c,e,g exhibited the measured spectra under different net intra-cavity dispersion, with corresponding autocorrelation traces shown in Figure 3b,d,f,h, respectively. The autocorrelation traces were sech^2^ fitted. Under −0.021, −0.016, −0.008, and −0.002 ps^2^ for the net dispersion, the central wavelength for the output spectra were 1029, 1035, 1040, and 1042 nm, with 145.8, 138.0, 110.8, and 99.8 fs, for the corresponding pulse duration. The pulse operated without splitting.

When the net anomalous dispersion was rather large, the laser worked in the soliton-like regime. The optical spectrum showed a similar shape for the soliton pulse with Kelly sidebands but not exactly the same as the typical soliton pulse. It showed a multi-peak structure with a distinctive central peak. The modulation in the optical spectrum was due to the effect of self-phase modulation. As the separation of the grating pair decreased, the peaks started to fade in sequence from the sides to the center. In this process, the optical spectrum became broader, and the pulse duration went shorter. Near zero dispersion, the fiber laser-generated pulses with short pulse duration and a sideband-free, wide spectrum. In this case, the broadest spectrum was 12.06 nm at full width at half maximum (FWHM) when the grating separation was 9.5 mm, corresponding to a net cavity dispersion of −0.002 ps^2^. The corresponding pulse duration was only 99.8 fs assuming a sech^2^ shape. The calculated Fourier-transform limit of the optical spectrum was 94.6 fs, which was very close to the experimentally measured pulse duration.

**Figure 4:**
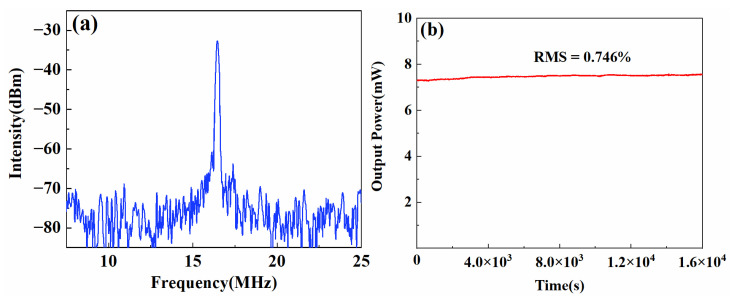
(**a**) RF spectrum at the net intra-cavity dispersion of −0.002 ps^2^. (**b**) Power fluctuation over 4.5 h.

The radiofrequency (RF) spectrum at the net intra-cavity dispersion of −0.002 ps^2^ is shown in Figure 4a. It was measured with 180 kHz resolution bandwidth and 20 MHz span by the same photodetector connected to an RF spectrum analyzer (KEYSIGHT, CXA Signal Analyzer 9 kHz-7.5 GHz, New Castle, DE, USA). The fundamental repetition rate of the pulse train was measured to be 16.03 MHz. The RF spectrum confirmed the stable operation of the mode-locking, with an absence of sidebands and harmonic frequencies. The ripple on both sides of the RF spectrum was due to the ringing of the photodetector. In Figure 4a, the signal-to-noise ratio (SNR) of the fundamental RF spectrum was 48 dB. To demonstrate the stability of the laser, the average output power of the output pulse was measured for a duration of 4.5 h. The pump power was set to 223 mW. The average output power was 7.47 mW. Figure 4b displayed the root mean square (RMS) as 0.746%. Owing to the use of the PM fiber, the stability of the fiber laser was mainly affected by the fluctuation of the pump power and the temperature.

## 4. Conclusions

As compared with previous approaches by using the figure-of-9 configuration, a 750 mW CW pump beam, delivered by a single-mode LD, was coupled into the fiber loop of the oscillator. In contrast, lower pump power (95–250 mW) was usually applied by earlier experiments on the figure-of-9 configuration [9,11,28,29] since pulse splitting could occur with strong intensity inside the cavity. In our case, pulse splitting was restrained by the dispersion management. This allowed the laser to operate under a high peak-power without splitting. Actually, in our experiment, the net inter-cavity dispersion was in the range of −0.021 to −0.002 ps^2^. Once the net dispersion was tuned out of the range, pulse splitting would happen or the mode-locking would disappear.

In conclusion, we demonstrated a figure-of-9 fiber laser, generating 99.8 fs near Fourier-transform-limit pulses with more than 25 kW peak-power. A stable mode-locked operation was achieved with dispersion management, with a maximum average power of 40.01 mW. Compared to the state-of-the-art Yb-doped PM-fiber ultrashort pulse oscillators with a figure-of-9 configuration, we achieved advances in pulse energy and peak-power of the direct output pulse. We believe the present ultrafast fiber oscillator could be an ideal laser source for various applications.

## Figures and Tables

**Table 1:** Comparison of direct output pulses of figure-of-9 mode-locked fiber lasers.

Ref	Spectral Width	Pulse Duration	Pulse Energy	Peak-Power
[26]	31 nm	61 fs	0.1 nJ	1.5 kW
[27]	7.8 nm	215 fs	0.21 nJ	0.4 kW
[28]	20 nm	1.86 ps	0.26 nJ	0.14 kW
[14]	14.1 nm	122 fs	0.61 nJ	4.85 kW
[13]	NA	64 fs	NA	10 kW
This work	12.06 nm	99.8 fs	2.5 nJ	25 kW

## Data Availability

Date are contained within the article.
